# Editorial: Characterizing Modern Microbialites and the Geobiological Processes Underlying Their Formation

**DOI:** 10.3389/fmicb.2019.02299

**Published:** 2019-10-09

**Authors:** Jamie S. Foster, R. Pamela Reid, Pieter T. Visscher, Christophe Dupraz

**Affiliations:** ^1^Department of Microbiology and Cell Science, Space Life Science Laboratory, University of Florida, Merritt Island, FL, United States; ^2^Rosenstiel School of Marine and Atmospheric Science, University of Miami, Miami, FL, United States; ^3^Department of Geosciences, University of Connecticut, Groton, CT, United States; ^4^Department of Geological Sciences, Stockholm University, Stockholm, Sweden

**Keywords:** microbialites, microbiome, geobiology, meta-omics approaches, microbiology

Microbialites represent one of the oldest known ecosystems on Earth, with a fossil record dating back over 3.5 billion years. These long-lived communities form sedimentary structures as a result of the synergy between microbial metabolisms and the environment. Although once global on the ancient Earth, modern microbialites are found mainly in restricted habitats with sparse eukaryotic populations. Living microbialites offer an opportunity to examine how these ancient ecosystems interface and respond to changes in their environment. Even today, microbialites are bellwethers for an ever-changing Earth and are becoming increasingly exposed to effects of global climate change, such as rising sea levels, ocean acidification, and warmer temperatures. Investigations into extant microbialites represent a unique opportunity to understand the feedbacks that occur between microbialite communities and their environment.

In this collection of research articles, experts investigate and discuss the formation of modern microbialites and the interactions between microbes and the environment. These research contributions target communities from a diverse range of freshwater, marine, and hypersaline environments. Key questions addressed by the papers include (Q1) what are the taxa and metabolic processes that influence microbialite formation? (Q2) How do microbes network and coordinate their activities to form lithified structures? (Q3) How do environmental conditions influence microbialite ecosystems both in the past and present? And (Q4) how are modern microbialite systems likely to respond to ongoing climate change?

Questions 1 and 2 are highly integrative and most papers in the collection touched upon these key areas of research. For example, Wilmeth et al. use novel tracer experiments to quantify mat biomass addition as well as assess the deposition of calcium carbonate within hot spring microbial mats. Their analysis of the rates of carbon fixation and biogenic carbonate precipitation suggests that metabolic processes other than autotrophy may play critical roles in the preservation of mats as microbialites. Additionally, Kraus et al. looked at the formation of minerals in microbialites to help improve our understanding of biosignature in hot springs. Their results reveal that abiotic mineralization of calcite can be subsequently modified by microbial activities, suggesting that biosignature formation is a complex, multi-stage process.

Also addressing Q1 and Q2, the role of certain phototrophic taxa in the formation of mineral precipitates was examined in diverse hypersaline environments including Laguna Negra in Argentina (Mlewski et al.) and Lake Dziani Dzaha on Mayotee Island (Gérard et al.), revealing a multifaceted role of phototrophs in microbialite precipitation. Additionally, several of the articles begin to characterize and close the genomes of some of the more abundant taxa within freshwater microbialites derived from Pavilion Lake in British Columbia (White et al.; White et al.), including novel species of the *Exiguobacterium* and *Agrococcus* genera. These efforts have helped expand the genome databases of taxa associated with the formation and growth of modern microbialites.

Although most studies on microbialites typically focus role of bacteria and archaea in the molecular and biochemical processes associated with element cycling and carbonate precipitation in microbialites, such as the study of Valdespino-Castillo et al., two studies within this collection also addressed Question 1 by targeting organisms that are typically overlooked—algae and viruses. In Frommlet et al., *in vitro* experiments using the alga *Symbiodinium* and its naturally associated microbial consortia revealed that bacterial-algal associations can affect the physicochemical macroenvironment in culture and that the structural integrity of the bacterial-algal biofilms in the microenvironment influences and can facilitate calcification. Alternatively, the role of viruses within microbialite-forming communities was explored by White et al. Their analysis revealed a diverse assemblage of single-stranded DNA viruses within the microbialites, which may be important in element cycling and perhaps modulating microbial diversity of microbialite communities.

In addition to examining microbialite formation, other authors explored Question 3 regarding the impact of the environment on microbialite-forming communities. In the paper by De Anda et al., the authors used metagenomic techniques to examine the specific interactions between taxa in response to environmental perturbations in the freshwater microbialites of Cuatros Ciénegas in Mexico. Their results show that water availability impacts the balance between competition and cooperation interactions. Similarly in the hypersaline system of Hamelin Pool in Western Australia, Babilonia et al. used comparative metagenomics to reveal different metabolic strategies for microbialite formation that was highly dependent on environment, in particular water depth.

Together, this collection of articles has provided new insight into the processes by which microbialites form and how these dynamic ecosystems potentially adapt to and alter their surrounding environment. These articles also reveal several universal processes associated with mineral precipitation across different habitats and help elucidate dynamic feedbacks that occur between microbialites and their environment. Although several of the manuscripts in this collection touch upon how modern microbialite systems are affected by ongoing changes in the climate, this last key question (Q4) represents an important frontier for microbialite research. As microbialites have persisted on Earth for most of evolutionary history, the mechanisms and pathways in which they have caused, responded and adapted to climate change represent a valuable resource to more fully explore the feedbacks and constraints underlying the continued habitability of our planet.

## Author Contributions

All authors listed have made a substantial, direct and intellectual contribution to the work, and approved it for publication.

### Conflict of Interest

The authors declare that the research was conducted in the absence of any commercial or financial relationships that could be construed as a potential conflict of interest.

